# The Role and Research Progress of CD8^+^ T Cells in Sepsis

**DOI:** 10.3390/biomedicines13122912

**Published:** 2025-11-27

**Authors:** Xianwen Wang, Qihang Huang, Zhihong Zuo, Zhanwen Wang, Lina Zhang, Zhaoxin Qian

**Affiliations:** 1 Department of Critical Care Medicine, Hunan Provincial Clinical Research Center for Critical Care Medicine, Xiangya Hospital, Central South University, Changsha 410008, China; 2 National Clinical Research Center for Geriatric Disorders, Xiangya Hospital, Central South University, Changsha 410008, China

**Keywords:** CD8^+^ T cells, sepsis, immune dysfunction, immune checkpoints, metabolic reprogramming

## Abstract

Sepsis is a systemic inflammatory response syndrome induced by infection, characterized by high morbidity and mortality, and responsible for over 11 million deaths worldwide annually. Recent studies have demonstrated that immune dysfunction represents a core element in the pathophysiology of sepsis, in which cluster of differentiation 8–positive (CD8^+^) T cells, as key executors of cellular immunity, play a critical role in immune dysregulation. This review systematically elaborates on the quantitative changes, functional status, and molecular regulatory mechanisms of CD8^+^ T cells in sepsis, including abnormalities in metabolic reprogramming, cell death pathways, transcriptional regulation, and intercellular communication. Additionally, it explores potential therapeutic strategies targeting CD8^+^ T cells, such as immune checkpoint modulation, cell death intervention, and metabolic regulation, and offers an outlook on future research directions, aiming to provide novel insights for immunotherapy in sepsis.

## 1. Introduction

Sepsis is one of the leading life-threatening critical illnesses worldwide, fundamentally arising from a dysregulated host response to infection [1]. Globally, there are approximately 50 million sepsis cases annually, with mortality exceeding 11 million, accounting for 20% of all global deaths [2]. The pathophysiology of sepsis is highly complex, involving a biphasic disturbance of early hyperinflammation and subsequent immunosuppression, wherein immune cell dysfunction persists throughout the disease course [3,4]. For a long time, research has predominantly focused on innate immunity and cytokine storms in sepsis, while adaptive immunity, especially cluster of differentiation 8–positive (CD8^+^) T cells, has received relatively insufficient attention.

CD8^+^ T cells, as the primary effector cells in cellular immunity, play a central role in clearing intracellular pathogens and mediating anti-tumor immunity [5,6]. In the septic milieu, CD8^+^ T cells not only undergo a marked numerical reduction but also exhibit profound alterations such as functional exhaustion and metabolic remodeling. Recent studies indicate that these abnormal changes in CD8^+^ T cells are closely associated with the extent of organ injury and prognosis in sepsis patients [7,8]. The application of novel technologies like single-cell sequencing enables exploration of the transcriptomic features and intercellular interactions of CD8^+^ T cells in sepsis at higher resolution, thereby revealing the intrinsic mechanisms underlying their functional dysregulation [9].

This review aims to systematically delineate the role of CD8^+^ T cells in the immunopathological mechanisms of sepsis, summarize recent advances in the functional regulation of CD8^+^ T cells, and discuss the developmental prospects of therapeutic strategies targeting CD8^+^ T cells, with the goal of providing a theoretical basis for improving immunotherapy in sepsis patients.

## 2. Abnormal Changes in CD8^+^ T Cells in Sepsis

### 2.1. Alterations in Quantity and Subsets

#### 2.1.1. Overall Quantitative Changes and Organ-Specific Distribution

A prominent feature of early sepsis is a sharp decline in total peripheral blood lymphocyte counts, known as lymphopenia, which extensively affects various T cell subsets, including CD8^+^ T cells [7,8,10]. Both clinical and animal studies consistently demonstrate significant reductions in CD4^+^ and CD8^+^ T cell numbers in the peripheral circulation and lymphoid organs such as the spleen [7,11]. Notably, different lymphocyte subsets exhibit varying sensitivities to sepsis-induced apoptotic signals; compared to B cells and CD4^+^ T cells, the numerical reduction in CD8^+^ T cells may be relatively less severe [12].

In stark contrast to the depletion of cells in the peripheral blood and lymphoid organs, there is a paradoxical accumulation of CD8^+^ T cells in specific organs such as the liver [11,13]. This phenomenon, termed “organ-specific cell sequestration,” highlights the complex regionalized characteristics of the immune response in sepsis. Regarding the mechanism behind CD8^+^ T cell accumulation in the liver, two main hypotheses exist: The “graveyard hypothesis” posits that activated CD8^+^ T cells entering the apoptotic program in the peripheral circulation are captured and cleared by hepatic macrophages (Kupffer cells) and sinusoidal endothelial cells, manifesting as temporary accumulation [13]. In contrast, the “hunting ground/killing field hypothesis” suggests that the liver, acting as an immune barrier organ, actively captures and “imprisons” activated T cells to restrict their potentially destructive systemic effects [13,14].

From a molecular perspective, the upregulation of adhesion molecules such as Intercellular Adhesion Molecule-1 (ICAM-1) on liver endothelial cells may mediate the adhesion and sequestration of CD8^+^ T cells within the liver sinusoids by binding to integrins on the T cell surface [13,14,15]. Furthermore, chemokine pathways like CXC Chemokine Receptor 3 (CXCR3) and its ligands (CXCL9, CXCL10) have been shown in other liver inflammation models to driveCD8^+^ T cell recruitment to the liver [16,17,18], suggesting this pathway might also play a role in hepatic T cell accumulation during sepsis, although its specific mechanisms require further clarification [13].

Accumulated CD8^+^ T cells in the liver can trigger hepatocyte apoptosis by expressing Fas ligand (FasL), which binds to Fas receptors on hepatocyte surfaces, thereby exacerbating liver dysfunction [13,19]. This CD8^+^ T cell-mediated cytotoxicity is an important mechanism of liver injury in sepsis-induced Multiple Organ Dysfunction Syndrome (MODS).

#### 2.1.2. Remodeling of Memory and Effector Subsets

Sepsis profoundly reshapes the internal subset composition of CD8^+^ T cells. Based on function and differentiation status, CD8^+^ T cells can be categorized into Naïve (Tn), Central Memory (Tcm), Effector Memory (Tem), and terminally differentiated Effector Memory (Temra) cells. Multiple studies report an imbalance in the peripheral blood CD8^+^ T cell population in both sepsis patients and animal models, characterized by a relative increase in the proportion of Central Memory T cells (Tcm) and a corresponding decrease in Effector Memory T cells (Tem) [20,21].

This shift towards a Tcm-dominant profile has significant functional implications. Although Tcm cells possess strong proliferative potential and long-term survival capacity, their immediate effector functions (such as cytokine production and direct killing) are relatively weak. In contrast, Tem cells exhibit rapid effector capabilities. Therefore, this remodeling of memory subsets likely impairs the body’s ability to promptly clear new or latent pathogens, representing an important manifestation of the immunosuppressed state.

Consistently, a recent review concluded that sepsis stochastically depletes naïve and circulating memory CD8^+^ T cells, while tissue-resident memory cells become vulnerable in more severe disease [22].

#### 2.1.3. Increase in Functionally Exhausted Subsets

T cell exhaustion, a common state of immune dysfunction in chronic infections and cancer, has recently been confirmed as a core feature of sepsis-induced immunosuppression [23]. CD8^+^ T cells from sepsis patients significantly upregulate the expression of multiple inhibitory receptors, including Programmed Death-1 (PD-1), T cell Immunoglobulin and Mucin-domain Containing-3 (TIM-3), and Lymphocyte Activation Gene-3 (LAG-3) [4,10,24].

These exhausted CD8^+^ T cells are not only functionally impaired themselves, but their presence is also associated with an increased risk of secondary infections and elevated mortality in patients with sepsis [25,26]. Recent high-resolution approaches such as single-cell sequencing have begun to dissect this phenomenon in much greater detail [27]. Single-cell multi-omics platforms that integrate transcriptomic profiles, T cell receptor repertoires, surface protein expression and, in some cases, epigenomic information can resolve multiple CD8^+^ T cell states along the continuum from effector to terminally exhausted phenotypes, quantify clonal expansion and lineage relationships, and link these states to clinical trajectories and organ dysfunction in sepsis [25,26,27]. When combined with spatial transcriptomics, these approaches further localize distinct CD8^+^ T cell subsets within tissue niches and reconstruct cell–cell communication networks with myeloid, stromal and endothelial cells in infected organs. Together, single-cell multi-omics and spatial profiling provide a powerful framework for mapping immune heterogeneity in sepsis and for identifying microenvironment-dependent vulnerabilities of CD8^+^ T cells that may be exploited therapeutically [27].

### 2.2. Functional Alterations

#### 2.2.1. Early Functional Response and Late Functional Exhaustion

The functional state of CD8^+^ T cells in sepsis exhibits dynamic characteristics. In the early stage of the disease, CD8^+^ T cells may retain certain cytotoxic functions, evidenced by the secretion of effector molecules (e.g., perforin, granzymes) and inflammatory cytokines (e.g., interferon-γ (IFN-γ), tumor necrosis factor-α (TNF-α)) [28,29,30]. However, as the disease progresses, CD8^+^ T cells gradually display features of functional exhaustion, characterized by a progressive loss of effector functions [10,31,32].

Functionally exhausted CD8^+^ T cells exhibit impairments in multiple key functions: reduced secretion of effector molecules, decreased capacity to produce inflammatory cytokines, and significantly diminished proliferative potential. Studies show that the direct contact-mediated killing function of CD8^+^ T cells is markedly impaired in sepsis patients, and indirectly mediated killing effects are also significantly reduced [33,34].

#### 2.2.2. Upregulation of Immune Checkpoint Molecules

The upregulation of immune checkpoint molecules is a hallmark of CD8^+^ T cell exhaustion. The expression of inhibitory receptors such as PD-1, cytotoxic T-lymphocyte–associated protein 4 (CTLA-4), and LAG-3 is significantly increased on CD8^+^ T cells from sepsis patients [35,36,37]. These molecules inhibit T cell activation by modulating downstream signaling pathways. Notably, different immune checkpoints may exhibit distinct regulatory patterns across various time windows and subsets.

High-level expression of PD-1, among others, is associated with the development of sepsis-induced Acute Respiratory Distress Syndrome (ARDS) and poor outcomes [25]. Studies also found that an increased PD-1/CD28 ratio on CD8^+^ T cells, rather than the PD-1 expression level alone, better predicts the risk of nosocomial infection in sepsis patients [38].

#### 2.2.3. Defective Metabolic Adaptability

Recent research has revealed that the functional dysregulation of CD8^+^ T cells in sepsis is also linked to defective metabolic adaptability [39]. CD8^+^ T cells fail to effectively adjust their metabolic programs to meet the energy and biosynthetic demands required for activation and effector functions. This metabolic defect further constrains their immune capacity. Among these alterations, abnormally enhanced glycolytic activity is closely associated with CD8^+^ T cell functional imbalance, and targeting this metabolic reprogramming process may restore CD8^+^ T cell function [40,41,42].

## 3. Mechanisms of CD8^+^ T Cell Dysfunction in Sepsis

In sepsis, CD8^+^ T cells are affected by several intertwined processes, including metabolic reprogramming, dysregulated cell death, altered transcriptional and epigenetic programs, and disturbed intercellular communication. Together, these cell-intrinsic and cell-extrinsic changes drive the numerical loss and functional paralysis of CD8^+^ T cells and sustain sepsis-induced immunosuppression.

To provide an overview of these heterogeneous alterations, Table 1 summarizes the key molecular changes observed in CD8^+^T cells during sepsis, including inhibitory checkpoint upregulation (e.g., PD-1), enhanced apoptosis signaling, metabolic and mitochondrial dysfunction, impaired effector molecule production, and transcriptional/epigenetic reprogramming.

### 3.1. Metabolic Reprogramming

In the septic environment, the metabolic network of CD8^+^ T cells undergoes substantial remodeling, which ultimately leads to energy failure and functional suppression. This process mainly involves mitochondrial dysfunction, abnormal glycolysis, and disruption of key metabolic regulatory pathways.

#### 3.1.1. Mitochondrial Dysfunction

During sepsis, mitochondria in CD8^+^ T cells are severely damaged, with characteristic impairment of mitochondrial respiration and redox balance [43,44,45]. Dysfunctional mitochondria produce insufficient adenosine triphosphate (ATP), directly limiting energy-intensive biological functions such as proliferation, cytokine synthesis, and cytotoxic granule release [46]. At the same time, they generate excessive reactive oxygen species (ROS), which cause oxidative damage to cellular components and further aggravate CD8^+^ T cell dysfunction [44]. In addition, increased mitochondrial membrane permeability promotes the release of pro-apoptotic factors such as cytochrome c, thereby activating the intrinsic apoptotic pathway [47,48,49].

**Table 1:** Key molecular alterations in CD8^+^ T cells during sepsis and their functional consequences.

Domain	Marker/Pathway	Change in Sepsis CD8^+^ T Cells	Functional Consequence	Main Evidence (Human/Murine/Other)	Immune Phase *	References
Immune checkpoint	PD-1, PD-L1	↑	Inhibits TCR signaling, proliferation, cytokine production	Human sepsis, murine CLP, chronic viral infection	Early → late	[12,35,36,37]
Immune checkpoint	CTLA-4, LAG-3, TIM-3, 2B4	↑	Synergistic inhibition, poly-checkpoint exhausted phenotype	Human sepsis, murine CLP, cancer models	Late	[18,38]
Cytokine production	IFN-γ, TNF-α	↓	Impaired macrophage activation and pathogen clearance	Human sepsis	Late	[20,43]
Survival/apoptosis	Bcl-2 (anti-apoptotic)	↓	Reduced survival, increased apoptosis	Human sepsis, murine CLP	Early	[8,21]
Survival/apoptosis	Fas/FasL, TRAIL, BAX/BAK	↑	Enhanced extrinsic and intrinsic apoptosis	Human sepsis, murine CLP	Early	[9,23,24]
Metabolism—glycolysis	GLUT1 expression	↑ or ↔	Compensatory upregulation but net glycolytic flux decreases in exhaustion	Chronic infection, cancer; limited sepsis data	Late	[46,47]
Metabolism—mitochondrial	Mitochondrial membrane potential, OXPHOS	↓	ATP shortage, ROS accumulation, release of cytochrome c	Human sepsis, murine models	Early → late	[25,48]
Oxidative stress	Mitochondrial & cytosolic ROS	↑	DNA/protein damage, activation of intrinsic apoptosis	Human sepsis, murine models	Early → late	[25,49]
Transcription factors	T-bet	↓	Loss of effector differentiation	Mainly murine infection, limited sepsis	Late	[50,51]
Transcription factors	EOMES, TOX	↑	Favors exhausted phenotype	Cancer and chronic infection models	Late	[52,53,54]
Epigenetics—DNA methylation	DNMT3a, global promoter methylation	↑	Stable silencing of effector and memory genes	Infection and cancer models	Late	[55,56]
Epigenetics—histone marks	EZH2 (H3K27me3), G9a (H3K9me2/3)	↑	Repressive chromatin at effector loci, maintained exhaustion	Sepsis (limited), chronic infection	Late	[57,58,59]

↑, increased/upregulated; ↓, decreased/downregulated; ↔, unchanged; * Immune phase: approximate timing (hyperinflammatory “early” vs. immunosuppressive “late”); PD-1, programmed cell death protein 1; PD-L1, programmed death-ligand 1; TCR, T-cell receptor;CLP, cecal ligation and puncture (standard murine model of polymicrobial sepsis); CTLA-4, cytotoxic T-lymphocyte–associated protein 4; LAG-3, lymphocyte activation gene-3; TIM-3, T-cell immunoglobulin and mucin-domain containing-3; 2B4, natural killer cell receptor 2B4 (CD244); IFN-γ, interferon-gamma; TNF-α, tumor necrosis factor-alpha; Bcl-2, B-cell lymphoma 2; Fas, Fas receptor (CD95); FasL, Fas ligand; TRAIL, TNF-related apoptosis-inducing ligand; BAX, Bcl-2–associated X protein; BAK, Bcl-2 homologous antagonist/killer; GLUT1, glucose transporter 1; OXPHOS, oxidative phosphorylation; ATP, adenosine triphosphate; ROS, reactive oxygen species; EOMES, eomesodermin; TOX, thymocyte selection–associated high-mobility-group box protein; DNMT3a, DNA (cytosine-5)-methyltransferase 3A; EZH2, enhancer of zeste homolog 2; H3K27me3, trimethylation of lysine 27 on histone H3; G9a, histone-lysine N-methyltransferase G9a; H3K9me2/3, di- or trimethylation of lysine 9 on histone H3; DNA, deoxyribonucleic acid.

#### 3.1.2. Abnormal Glycolytic Metabolism

Under physiological activation, CD8^+^ T cells rely on aerobic glycolysis (the Warburg effect) to support rapid proliferation and effector functions [40,50]. In sepsis-induced T cell exhaustion, however, exhausted CD8^+^ T cells cannot maintain glycolytic efficiency and flux at levels comparable to effector T cells, even though they may upregulate glucose transporters such as glucose transporter 1 (GLUT1) [51]. This impaired glycolytic capacity prevents cells from meeting their bioenergetic and biosynthetic demands, driving them into a low-energy “dormant” or exhausted state [40,52].

#### 3.1.3. Disruption of Key Metabolic Regulatory Pathways

Multiple signaling pathways cooperate to regulate the metabolic state of CD8^+^ T cells. Persistent activation of the PD-1 pathway inhibits phosphatidylinositol 3-kinase –Akt–mammalian target of rapamycin (PI3K–Akt–mTOR) signaling, a central driver of glycolysis and mitochondrial metabolism; consequently, high PD-1 expression directly contributes to metabolic suppression in CD8^+^ T cells [52]. In addition, molecules such as sirtuin 1 (SIRT1) and peroxisome proliferator-activated receptor-β/δ (PPARβ/δ), which are important for guiding CD8^+^T cells toward a memory phenotype and maintaining metabolic flexibility, may also become dysregulated in the complex septic milieu [53,54].

These metabolic constraints not only blunt effector functions but also render CD8^+^ T cells more susceptible to programmed cell death. Thus, metabolic failure provides a permissive background for the activation of apoptotic pathways, thereby accelerating the numerical loss of CD8^+^ T cells in sepsis.

### 3.2. Abnormal Cell Death Pathways

The marked loss of CD8^+^T cells in sepsis is mainly mediated by programmed cell death, particularly through aberrant activation of both extrinsic and intrinsic apoptotic pathways.

#### 3.2.1. Extrinsic Apoptotic Pathway

The extrinsic pathway is initiated by cell-surface death receptors, among which the Fas/FasL axis is the best-characterized mechanism of CD8^+^ T cell apoptosis in sepsis. In this setting, CD8^+^ T cells upregulate Fas ligand (FasL), whereas Fas expression is increased on target cells such as hepatocytes [13]. Engagement of Fas by FasL recruits the Fas-associated death domain (FADD), activates caspase-8 and the downstream caspase cascade, and ultimately drives apoptosis; TNF-related apoptosis-inducing ligand (TRAIL) uses similar receptor–ligand interactions to promote lymphocyte death in sepsis [55,56,57,58].

#### 3.2.2. Intrinsic Apoptotic Pathway

The intrinsic pathway is triggered by intracellular stress and is critically regulated by the B-cell lymphoma-2 (Bcl-2) family [47,48,59]. In sepsis, severe inflammatory and metabolic stress tilts this balance toward pro-apoptotic members such as Bcl-2-associated X protein (Bax) and Bcl-2 homologous antagonist/killer (Bak), while anti-apoptotic proteins (e.g., Bcl-2, Bcl-xL) are downregulated [48,60]. Bax/Bak-mediated permeabilization of the mitochondrial outer membrane allows for cytochrome c release and formation of the apoptosome, which activates caspase-9 and converges on the common executioner caspase-3 [47,60,61].

#### 3.2.3. Apoptotic Execution Phase

Caspase-8 from the extrinsic pathway and caspase-9 from the intrinsic pathway converge on the activation of the effector caspase-3, which then cleaves structural and nuclear proteins to dismantle the cell and complete apoptosis [47,56,62].

Importantly, apoptosis does not fully explain CD8^+^ T cell dysfunction in sepsis. Even among surviving cells, sepsis leaves a long-lasting “imprint” in the form of altered transcriptional and epigenetic programs that stabilize the exhausted phenotype.

### 3.3. Transcriptional Regulation and Epigenetic Alterations

The durable impact of sepsis on CD8^+^ T cell function is closely linked to re-wiring of transcription factor networks and epigenetic landscapes, which together lock T cells into a hyporesponsive state.

#### 3.3.1. Disruption of the Transcription Factor Network

The differentiation and function of CD8^+^ T cells are orchestrated by a coordinated transcription factor network. In sepsis and other chronic inflammatory conditions, the balance between effector- and exhaustion-associated transcription factors is shifted. Downregulation of T-bet and preferential upregulation of exhaustion-related factors such as eomesodermin (EOMES) push CD8^+^ T cells away from a robust effector fate toward a dysfunctional, exhausted state, characterized by sustained expression of inhibitory receptors and limited cytokine production [22].

#### 3.3.2. DNA Methylation Changes

DNA methylation is a key epigenetic modification associated with stable transcriptional repression. In sepsis, expression of the DNA methyltransferases DNA methyltransferase 1 (DNMT1), DNA methyltransferase 3a (DNMT3a) and DNA methyltransferase 3b (DNMT3b) is increased [63]. DNMT3a in particular shapes the exhausted CD8^+^ T cell program by methylating promoters and regulatory regions of effector and memory-associated genes such as transcription factor 7 (Tcf7), while demethylation at loci encoding inhibitory receptors (e.g., PD-1) stabilizes their high-level expression [64,65,66,67,68,69].

#### 3.3.3. Histone Modification Regulation

Histone modifications dynamically regulate gene accessibility and expression by altering chromatin structure. In sepsis-associated CD8^+^ T cell exhaustion, an imbalance between histone acetyltransferases (HATs) and deacetylases (HDACs) may favor deacetylation at effector gene loci, leading to chromatin condensation and reduced transcription [70,71,72]. In parallel, increased activity of histone methyltransferases such as enhancer of zeste homolog 2 (EZH2; mainly targeting histone H3 lysine 27, H3K27) and G9a (primarily targeting histone H3 lysine 9, H3K9) deposits repressive marks—including trimethylation of histone H3 lysine 27 (H3K27me3) and di- or trimethylation of histone H3 lysine 9 (H3K9me2/3)—at effector and memory-related genes, thereby stabilizing the exhausted phenotype; pharmacologic inhibition of these enzymes can partially restore T cell function and downregulate PD-1 expression [73,74,75,76].

These transcriptional and epigenetic changes are further reinforced by an altered extracellular milieu. In the following section, we summarize how dysregulated intercellular communication—through inhibitory receptor–ligand interactions and cytokine networks—stabilizes CD8^+^ T cell exhaustion in sepsis.

### 3.4. Altered Intercellular Communication

The function of CD8^+^ T cells highly depends on precise communication with other immune cells and target cells; this communication network becomes disrupted in sepsis.

#### 3.4.1. Enhanced Inhibitory Receptor/Ligand Interactions

Under persistent antigen and inflammatory stimulation, CD8^+^ T cells highly express various co-inhibitory receptors. The PD-1/PD-L1 axis is the most classical mechanism of T cell exhaustion. CD8^+^ T cells from sepsis survivors persistently express high levels of PD-1 [77,78,79]. When PD-1 binds to its ligand PD-L1, which is widely expressed on antigen-presenting cells (APCs) or tissue cells, it delivers a strong inhibitory signal into the T cell, blocking TCR signal transduction and inhibiting cell proliferation, cytokine production, and cytotoxicity [80,81]. Besides PD-1, the expression of other inhibitory receptors such as LAG-3 and 2B4 on CD8^+^ T cells in sepsis is also significantly increased; their binding with respective ligands synergistically enhances the inhibition of T cell function [77,82,83].

#### 3.4.2. Shift in Cytokine Communication Patterns

While a “cytokine storm” may occur in the early stages of sepsis, the cytokine communication capacity of CD8^+^ T cells becomes severely impaired during the subsequent immunosuppressive phase. CD8^+^ T cells that survive sepsis show a significantly reduced ability to produce key effector cytokines such as IFN-γ and TNF-α upon re-encountering antigen [24]. IFN-γ is a core cytokine for activating macrophages, enhancing antigen presentation, and exerting anti-viral/intracellular bacterial effects; its reduced secretion implies that CD8^+^ T cells cannot effectively coordinate other immune cells, leading to a collapse of overall immune defense capability.

#### 3.4.3. Other Modes of Communication

Regarding other modes of intercellular communication, such as material and information exchange via exosomes or gap junctions, existing research evidence on their impact on CD8^+^ T cell function in the context of sepsis is limited, representing an area requiring further exploration.

**Figure 1:**
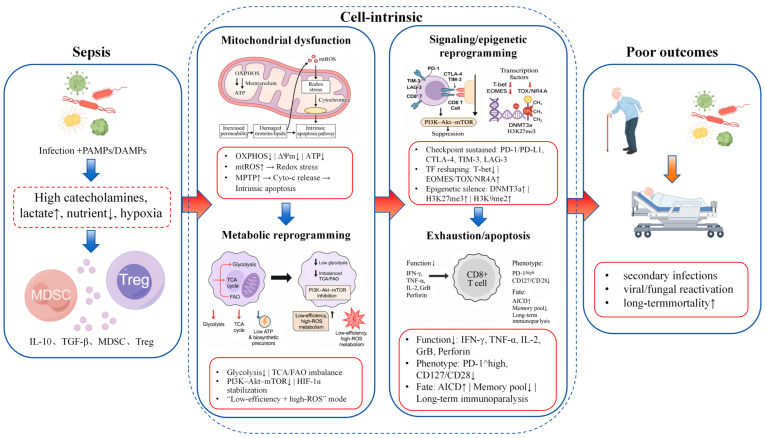
Integrated schematic of sepsis-induced CD8^+^ T cell dysfunction. Sepsis triggers systemic inflammation and metabolic stress, exposing CD8^+^ T cells to high levels of pathogen-associated molecular patterns (PAMPs) and damage-associated molecular patterns (DAMPs), catecholamines, hypoxia, lactate accumulation and nutrient deprivation. These insults, together with suppressive cues from myeloid-derived suppressor cells (MDSCs), regulatory T cells (Tregs) and immunosuppressive cytokines such as interleukin-10 (IL-10) and transforming growth factor-β (TGF-β), promote mitochondrial dysfunction characterized by impaired oxidative phosphorylation (OXPHOS), loss of mitochondrial membrane potential, reduced adenosine triphosphate (ATP) production and excessive generation of mitochondrial reactive oxygen species (mtROS). Mitochondrial insufficiency and redox stress act as upstream triggers of maladaptive metabolic reprogramming, including defective glycolysis, tricarboxylic acid (TCA) cycle and fatty acid oxidation (FAO), as well as inhibition of phosphatidylinositol 3-kinase (PI3K)–Akt–mammalian target of rapamycin (mTOR) signaling. These metabolic derangements feed into altered transcriptional and epigenetic programs, with downregulation of effector-associated transcription factors such as T-bet and upregulation of exhaustion-related factors including eomesodermin (EOMES), thymocyte selection-associated high-mobility-group box protein (TOX) and nuclear receptor subfamily 4 group A (NR4A) family members, accompanied by increased DNA methylation and repressive histone marks at effector gene loci. In parallel, persistent engagement of immune checkpoints, including programmed cell death protein 1 (PD-1) and its ligand programmed death-ligand 1 (PD-L1), cytotoxic T lymphocyte-associated protein 4 (CTLA-4), T-cell immunoglobulin and mucin-domain containing-3 (TIM-3) and lymphocyte activation gene-3 (LAG-3), further dampens T-cell receptor signaling and co-stimulatory pathways. Together, these cell-intrinsic and cell-extrinsic processes culminate in CD8^+^ T cell exhaustion and apoptosis, reflected by reduced cytokine production, diminished cytotoxic granule content, loss of proliferative capacity, contraction of the memory pool and increased susceptibility to secondary infections and late mortality.

To provide an integrated overview of these pathways, we created a schematic illustration that summarizes how sepsis-associated systemic inflammation and metabolic stress induce mitochondrial dysfunction, drive maladaptive metabolic reprogramming and ultimately converge on CD8^+^ T cell exhaustion and apoptosis (Figure 1). The figure highlights the central role of impaired oxidative phosphorylation and excessive mitochondrial reactive oxygen species (mtROS) production as upstream triggers of metabolic failure and redox stress, the downstream impact on transcriptional and epigenetic programs that stabilize the exhausted phenotype, and the contribution of extrinsic signals such as immune checkpoint engagement and immunosuppressive cytokines to sustaining CD8^+^ T cell dysfunction.

## 4. Therapeutic Strategies Targeting CD8^+^ T Cells

### 4.1. Immune Checkpoint Modulation

Immune checkpoint modulation aims to reverse the functional exhaustion of CD8^+^ T cells in sepsis. T cell exhaustion represents one of the core mechanisms underlying immunosuppression in sepsis, with its molecular basis rooted in the persistent activation of immune checkpoint signaling pathways. Under normal physiological conditions, these pathways serve to prevent excessive immune responses; however, in chronic inflammatory contexts such as sepsis, they inhibit CD8^+^ T cell activation, proliferation, and cytokine secretion.

PD-1 and its ligand (PD-L1) constitute the best-characterized immune checkpoint axis in sepsis. In septic patients, PD-1 expression is significantly upregulated on CD8^+^ T cells, while PD-L1 expression is concurrently increased on tissue parenchymal cells and antigen-presenting cells; their interaction potently suppresses T cell function and is associated with increased secondary infections, organ dysfunction and mortality [84,85]. Preclinical studies demonstrate that blocking this pathway with anti-PD-1 or anti-PD-L1 antibodies can restore the capacity of CD8^+^ T cells to produce IFN-γ and interleukin-2 (IL-2), enhance bacterial clearance in several cecal ligation and puncture (CLP) model and acute liver injury models, reduce lymphocyte apoptosis and improve survival rates [85,86]. However, not all experimental data are uniformly positive. In certain pneumonia models, anti-PD-L1 therapy reduced PD-L1 expression and altered inflammatory profiles but failed to improve survival [87,88]. In a cancer–sepsis model, PD-1 blockade did not confer a survival advantage and was associated with a loss of CXCR5^+^PD-1^+^ T cells and decreased CD28 expression, whereas blockade of another checkpoint receptor (2B4) significantly improved outcomes [89]. A recent systematic review and meta-analysis of checkpoint inhibitor therapy in preclinical sepsis models, as well as newer work in experimental bacterial infection, similarly indicates that although PD-1/PD-L1 inhibition improves bacterial control and survival in many settings, a substantial proportion of studies report neutral effects and a minority even demonstrate worsened outcomes, highlighting the importance of pathogen type, infectious focus, underlying comorbidities, dosing and timing of intervention when interpreting these data [90,91].

Beyond the PD-1/PD-L1 axis, other immune checkpoint molecules such as LAG-3 also contribute to T cell suppression in sepsis. Research indicates that LAG-3 and PD-1 are co-upregulated on T cells from sepsis patients, and they act synergistically to inhibit CD8^+^ T cell function [37]. The double-positive (LAG-3^+^PD-1^+^) CD8^+^ T cell subset exhibits severely impaired proliferative capacity, and its proportion is closely associated with adverse patient outcomes [37].Consequently, combined blockade of multiple immune checkpoint pathways (e.g., PD-1 and LAG-3) may offer superior therapeutic potential compared to single-target approaches.

Although preclinical findings are encouraging, the clinical translation of PD-1/PD-L1–directed immune checkpoint inhibitors (ICIs) in sepsis still faces substantial challenges. First, commonly used animal models differ widely in age, genetic background, pathogen type, infectious focus and supportive care, which contributes to heterogeneous and sometimes conflicting results and limits extrapolation to the highly heterogeneous human sepsis population [90,92]. Second, most preclinical studies have been conducted in otherwise healthy young mice, whereas real-world sepsis frequently occurs in older patients with multiple comorbidities, preexisting malignancy or prior exposure to ICIs, all of which may profoundly modify the risk–benefit profile of checkpoint blockade [93,94]. Third, early-phase clinical trials of PD-1/PD-L1 blockade in sepsis have so far involved small cohorts and primarily assessed immunological surrogates (e.g., restoration of ex vivo cytokine production) rather than hard clinical endpoints such as mortality or long-term functional recovery [95,96,97]. Finally, the safety profile of ICIs in critically ill patients, particularly the risk of immune-related adverse events (irAEs) such as pneumonitis, colitis or myocarditis, remains a major concern and underscores the need for careful patient selection, biomarker-guided timing and dose optimization [93,98].

Notably, sepsis-induced CD8^+^ T cell exhaustion closely parallels the exhaustion programs described in chronic infections and cancer. In these settings, persistent antigen and inflammation drive a spectrum of exhausted states, from T-cell factor 1–positive (TCF1⁺) cells with retained proliferative capacity to terminally exhausted thymocyte selection–associated high-mobility group box protein-positive (TOX⁺) cells with fixed epigenetic “scars” and limited responsiveness to checkpoint blockade [64,65,66,67]. Similar transcriptional, epigenetic and metabolic features have been reported in exhausted CD8^+^ T cells from septic patients, suggesting that principles derived from cancer immunotherapy may be directly applicable to sepsis [32,64,65,66,67,99,100]. For example, experience in oncology indicates that effective PD-1/PD-L1 blockade depends on the presence of a progenitor exhausted pool, that combined targeting of multiple checkpoints (e.g., PD-1 plus LAG-3 or CTLA-4) can overcome resistance in selected patients, and that integrating checkpoint inhibitors with cytokines, metabolic modulators or epigenetic drugs can further reinforce T cell reinvigoration [66,75,99,100,101,102,103]. Adapting these cross-disciplinary insights to the septic context may help refine patient selection, optimize treatment timing, and design rational combination regimens that more effectively restore CD8^+^ T cell function while limiting toxicity. In an “immunologically experienced” murine model, CD28 agonism improved CLP sepsis survival in a CD8^+^ T cell–dependent manner and dampened systemic cytokine release [104].

Two early-phase clinical trials have provided the first translational evidence for targeting the PD-1/PD-L1 axis in human sepsis. In a phase I dose-escalation study of the anti–PD-L1 antibody BMS-936559 in patients with severe sepsis and lymphopenia, single-dose administration was well tolerated, without evidence of drug-induced hypercytokinemia or secondary “cytokine storm”; at the two highest doses, treatment increased monocyte human leukocyte antigen–DR (HLA-DR) expression and improved ex vivo T cell function, although the study was not powered to detect a survival benefit [105]. Similarly, a phase Ib randomized trial of the anti–PD-1 antibody nivolumab in septic patients with features of immunosuppression demonstrated acceptable safety, sustained PD-1 receptor occupancy at relatively low doses, and enhancement of immune biomarkers such as interferon-γ production, again without definitive effects on mortality [88]. Pharmacokinetic and modeling work further suggests that lower doses than those used in oncology may be sufficient to restore immune competence in critically ill patients, potentially reducing the risk of immune-related adverse events [89]. Together, these studies indicate that checkpoint inhibition is clinically feasible in carefully selected septic patients and can reverse some hallmarks of immune paralysis, but they also underscore that current evidence remains confined to small, early-phase trials with surrogate endpoints rather than hard clinical outcomes.

### 4.2. Intervention in Cell Death Pathways

During sepsis, massive lymphocyte apoptosis directly contributes to the sharp decline in CD8^+^ T cell numbers and the impairment of immune function. Therefore, intervening in cell death pathways to preserve lymphocyte counts has emerged as a vital therapeutic strategy.

Sepsis-induced T cell apoptosis involves multiple molecular mechanisms, including the extrinsic pathway mediated by Fas/FasL and TNF-related apoptosis-inducing ligand (TRAIL), as well as the intrinsic mitochondrial pathway [58,106]. Among anti-apoptotic strategies, the application of cytokines that promote lymphocyte survival and proliferation shows considerable promise.

Among anti-apoptotic strategies, the application of common γ-chain cytokines that promote lymphocyte survival and proliferation shows considerable promise. Interleukin-7 (IL-7), a key lymphopoietic factor, inhibits lymphocyte apoptosis by upregulating the anti-apoptotic protein Bcl-2 and supporting homeostatic proliferation. In oncology and HIV infection, multiple trials of the glycosylated recombinant human IL-7 CYT107 have demonstrated a favorable safety profile and a consistent 2–4-fold increase in circulating CD4^+^ and CD8^+^ T cell counts, together with improved T cell function [90,107]. Importantly, the IRIS-7 randomized, double-blind, placebo-controlled trial in patients with septic shock and profound lymphopenia showed that intramuscular CYT107 was safe, induced a 3–4-fold expansion of circulating CD4^+^ and CD8^+^ T cells, and enhanced ex vivo interferon-γ production without causing sustained hemodynamic instability; the main adverse events were injection-site reactions and transient pyrexia associated with modest cytokine elevations [107]. Subsequent studies evaluating intravenous administration of CYT107 confirmed its biological activity in reversing sepsis-induced lymphopenia but also reported more pronounced transient cytokine peaks, suggesting that the intramuscular route may offer a more favorable balance between efficacy and tolerability in this fragile population [91]. Ongoing and recently completed multicenter phase II trials are further exploring different dosing schedules and routes of administration (e.g., NCT02640807, NCT03821038), as well as the impact of IL-7–mediated lymphocyte restoration on clinically relevant outcomes; however, these studies remain relatively small and have not yet established a clear survival benefit in sepsis [91,92].

Cellular autophagy is a crucial mechanism for maintaining intracellular homeostasis, and its dysfunction is closely linked to apoptosis. Studies in a lethal candidal sepsis model revealed that the mammalian target of rapamycin (mTOR) signaling pathway is hyperactivated in CD8^+^ T cells, leading to impaired autophagy flux and subsequently exacerbating apoptosis [108]. Suppressing this pathway through T cell-specific mTOR gene knockout restored autophagy, reduced CD8^+^ T cell apoptosis, and significantly improved survival in infected mice [108]. These results suggest that targeting the mTOR-autophagy axis (e.g., using rapamycin or analogous agents) could emerge as a novel strategy to protect CD8^+^ T cells and counteract sepsis-induced immunosuppression.

### 4.3. Metabolic Regulation Therapy

Cellular metabolism is tightly linked to the functional status of immune cells, a field referred to as immunometabolism. CD8^+^ T cells undergo profound metabolic reprogramming during activation, differentiation and memory formation [75,99,100]. In sepsis, systemic metabolic disturbances and a deteriorated microenvironment—characterized by hyperglycemia, lactate accumulation and nutrient depletion—severely compromise the glycolytic and mitochondrial fitness of CD8^+^ T cells, promoting oxidative stress and functional exhaustion [25,100,109]. Although preclinical studies directly targeting CD8^+^ T cell metabolism in sepsis are still limited, insights from immuno-oncology and chronic infections suggest that rational metabolic interventions may help restore T cell function.

To address these metabolic challenges, future investigations could explore several intervention strategies:

(1) Optimizing energy supply: Exogenous supplementation of key metabolic substrates such as glutamine, acetate, or ketone bodies may provide alternative energy sources for T cells, bypassing compromised metabolic pathways [101].
(2) Remodeling metabolic pathways: Small molecule drugs targeting critical metabolic enzymes or signaling pathways could be utilized. For instance, moderate inhibition of glycolysis might promote the conversion of exhausted T cells toward a memory phenotype [102,103]; regulating mitophagy to clear damaged mitochondria and restore oxidative phosphorylation capacity represents another promising approach.
(3) Targeted delivery systems: The lack of cellular specificity poses a major challenge for metabolic interventions. Systemic administration may induce side effects [101], underscoring the need for precise delivery systems such as antibody-drug conjugates (ADCs) or nanocarrier technologies to specifically transport metabolic modulators to CD8^+^ T cells [110].

Many of these metabolic perturbations in sepsis mirror those encountered by CD8^+^ T cells within the tumor microenvironment, where competition for glucose, amino acids and lipids, as well as hypoxia and acidosis, drive a similar trajectory toward functional exhaustion [78,96,97]. In cancer immunotherapy, a growing body of work has shown that fine-tuning T cell metabolism—for example, by moderately restricting glycolysis to favor memory-like differentiation, enhancing fatty acid oxidation, or using “metabolic immunoengineering” strategies—can significantly augment the efficacy and durability of CD8^+^ T cell–based therapies and immune checkpoint blockade [96,99,100,101]. These cross-disciplinary advances suggest that rationally designed metabolic interventions, guided by immune-metabolic profiling, may likewise be leveraged to rescue exhausted CD8^+^ T cells in sepsis and to synergize with cytokine therapy or checkpoint modulation.

### 4.4. Other Innovative Therapeutic Strategies

Advances in biotechnology have enabled innovative strategies such as cell therapy and gene editing, offering new avenues for enhancing CD8^+^ T cell function in sepsis treatment.

Adoptive cell therapy (ACT), although not yet directly applied in sepsis, possesses a core concept—the in vitro modification and reinfusion of functionally enhanced T cells—that holds significant inspirational value. Potential applications include:

(1) “Exhaustion-resistant” T cells: Employing gene editing technologies like clustered regularly interspaced short palindromic repeats and associated protein 9 (CRISPR/Cas9) [111,112] to knockout immune checkpoint genes (e.g., PD-1, LAG-3) in CD8^+^ T cells from sepsis patients, thereby enabling the reinfused cells to resist the inhibitory microenvironment and sustain cytotoxic activity.
(2) Pathogen-specific T cells: For sepsis caused by specific pathogens such as drug-resistant bacteria or fungi, CD8^+^ T cells capable of efficiently recognizing these pathogens can be isolated, expanded, or engineered via T cell receptor (TCR) gene modification, achieving “cellular antibiotic”-like precision therapy.

Furthermore, sepsis can induce lasting epigenetic alterations in T cells, locking them into a functionally suppressed “imprinted” state [113,114]. Utilizing epigenetic modulators, such as histone deacetylase (HDAC) inhibitors or DNA methylation inhibitors, to “reprogram” exhausted CD8^+^ T cells, reverse inhibitory gene silencing, and restore effector functions represents another innovative direction worthy of exploration. Similar epigenetic mechanisms underlie CD8^+^ T cell exhaustion in cancer, where stable DNA methylation and repressive histone marks at effector loci limit the durability of responses to checkpoint inhibitors and adoptive cell therapies [67,68,69,72,79]. Preclinical and early clinical studies in oncology have demonstrated that combining epigenetic modulators with ICIs or engineered T cells can partially erase exhaustion-associated epigenetic programs and improve antitumor efficacy [67,68,69,79]. These findings provide a strong rationale to explore analogous epigenetic “reprogramming” strategies for reversing sepsis-induced CD8^+^ T cell exhaustion.

## 5. Summary and Outlook

This review has systematically elaborated on the central role of CD8^+^ T cells in the immunopathological mechanism of sepsis and their potential as therapeutic targets. In sepsis, CD8^+^ T cells undergo significant numerical reduction, imbalance in subset proportions, and functional exhaustion, changes which are closely associated with patient prognosis. At the mechanistic level, metabolic reprogramming, dysregulated cell death, altered transcriptional regulation, and disrupted intercellular communication collectively underpin CD8^+^ T cell dysfunction.

Limitations and controversies in current evidence:

While the present review summarizes converging evidence that CD8^+^ T cells are numerically depleted and functionally exhausted in sepsis and that targeted interventions hold promise for restoring immunity, it is equally important to acknowledge the limitations and controversies of the current literature. First, most mechanistic data are derived from preclinical models or small single-center cohorts, with substantial variability in sepsis etiology, timing of sampling, immunophenotyping strategies and endpoints, which may partially explain discrepancies between studies. Second, for immune checkpoint blockade, cytokine supplementation (e.g., IL-7, IL-15) and metabolic or epigenetic interventions, the majority of studies have focused on short-term immunological readouts rather than long-term clinical outcomes, and several well-designed animal studies have reported neutral or even detrimental effects under specific conditions. Third, the exhausted phenotype itself may have a context-dependent protective component by limiting immunopathology during the hyperinflammatory phase, raising the possibility that indiscriminate reversal of exhaustion could exacerbate tissue damage in certain patients. Finally, older individuals and those with comorbidities such as cancer, chronic infections or prior exposure to immunotherapy are underrepresented in existing datasets, yet these groups are highly relevant to real-world sepsis and may respond differently to CD8^+^ T cell-targeted therapies.

Future research should be deepened in the following aspects:

(1) Utilize single-cell multi-omics technologies (such as scRNA-seq, scATAC-seq, TCR sequencing and CITE-seq) combined with spatial transcriptomics to comprehensively map CD8^+^ T cell states, clonal architecture, epigenetic programs and cell–cell interaction networks in blood and tissues across different stages of sepsis. Such integrated, spatially resolved datasets will refine current immune subtyping schemes, clarify how local microenvironments drive CD8^+^ T cell exhaustion, and provide a rational basis for designing CD8^+^ T cell-targeted interventions.
(2) Conduct in-depth investigation into memory formation and the long-term immunological impacts of CD8^+^ T cells in sepsis, particularly their defensive capacity against secondary infections.
(3) Systematically explore the cross-disciplinary interface between sepsis-induced CD8^+^ T cell exhaustion and cancer immunotherapy. On the one hand, studies of exhausted tumor-infiltrating CD8^+^ T cells have delineated hierarchical exhaustion states, key transcriptional/epigenetic regulators and metabolic checkpoints, and have identified biomarkers that predict responsiveness or resistance to immune checkpoint inhibitors [67,68,69,70,96,97,99,100,101]. Applying these conceptual frameworks and analytical tools (e.g., single-cell multi-omics, exhaustion state scoring, epigenetic profiling) to sepsis may help distinguish reversible from terminally fixed exhaustion, optimize checkpoint-based or adoptive T cell therapies, and inspire rational combination strategies. On the other hand, severe infections and sepsis episodes may reshape the CD8^+^ T cell compartment and its epigenetic “imprinting” in patients with cancer, thereby influencing subsequent responses and toxicity profiles to immunotherapies. Clarifying these bidirectional interactions will be essential for designing safer and more effective immunomodulatory regimens in both critical care and oncology settings.
(4) Develop precise immunomodulatory strategies targeting CD8^+^ T cells, including specific metabolic interventions, blockade of cell death pathways, and epigenetic remodeling.
(5) Promote the clinical translation process of innovative drugs (such as STC3141), addressing the current gap in targeted therapies for sepsis.

Despite the immense challenges, with the deepening understanding of sepsis immunology and the emergence of new technologies, therapeutic strategies targeting CD8^+^ T cells are expected to achieve major breakthroughs in the next decade, paving new avenues for improving the prognosis of sepsis patients.

## Data Availability

No new data were created or analyzed in this study. Data sharing is not applicable to this article.
